# Crumble analysis of the historic sympatric distribution between *Dendrortyx macroura* and *D*. *barbatus* (Aves: Galliformes)

**DOI:** 10.1371/journal.pone.0183996

**Published:** 2017-09-01

**Authors:** Claudio Mota-Vargas, Jorge Galindo-González, Octavio R. Rojas-Soto

**Affiliations:** 1 Instituto de Biotecnología y Ecología Aplicada (INBIOTECA), Universidad Veracruzana, Xalapa, Veracruz, México; 2 Laboratorio de Bioclimatología, Red de Biología Evolutiva, Instituto de Ecología A.C., Xalapa, Veracruz, México; Sichuan University, CHINA

## Abstract

In Mexico, the Long-tailed Wood-Partridge (*Dendrortyx macroura*) is distributed in the mountains of the Trans-Mexican Volcanic Belt, Sierra Madre del Sur and Sierra Norte de Oaxaca; while the Bearded Wood-Partridge (*D*. *barbatus*) is distributed in the Sierra Madre Oriental (SMO). There is a controversial overlap in distribution (sympatry) between these two species (on the Cofre de Perote and Pico de Orizaba volcanoes, SMO and Sierra Norte de Oaxaca), based on the ambiguity and current lack of information regarding the distribution of these two species. In order to disentangle the possible presence of both species in the area of sympatry, we conducted a crumble analysis of the historic knowledge regarding the geographic distribution of both species, based on a review of scientific literature, database records, the specimen examination (in ornithological collections), field work and a reconstruction of the distribution range based on Ecological Niche Modeling. Our results support the presence of only one of these two species in the overlapping area, rejecting the existence of such an area of sympatry between the two species. We discuss alternative hypotheses that could explain the historically reported distribution pattern: 1) an error in the single existing historical record; 2) a possible local extinction of the species and 3) the past existence of interspecific competition that has since been resolved under the principle of competitive exclusion. We propose that the Santo Domingo River in northern Oaxaca and western slope of the Sierra Madre Oriental, mark the distribution limits between these species.

## Introduction

**Fig 1:**
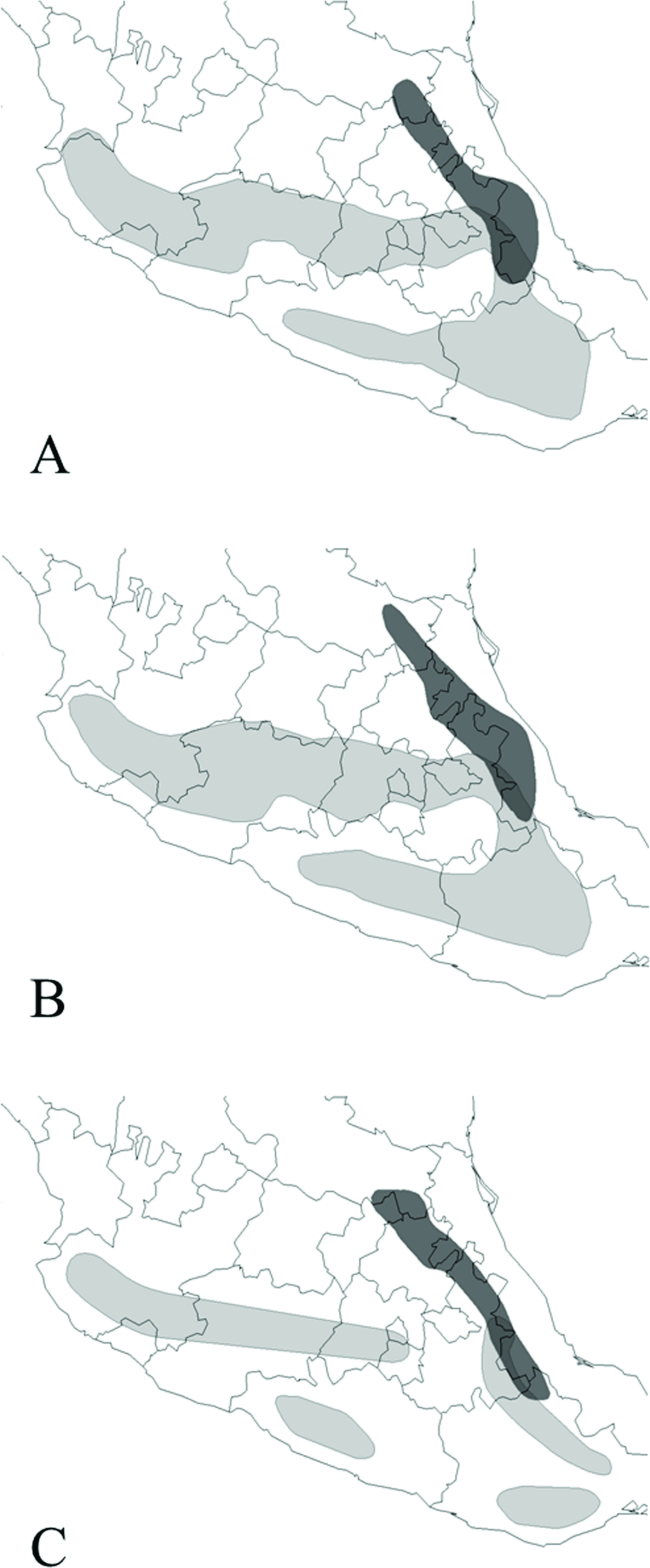
**Our representation of the distribution and supposed area of overlap between *D. macroura* (light gray) and *D. barbatus* (dark gray), according to different authors and dates AS Leopold 1959, A; PA Johnsgard 1973, B; And SN Howell & S Webb 1995, C**.

The wood partridge genus *Dendrortyx* (Galliformes, Odontophoridae) includes three species: *D*. *leucophrys*, *D*. *macroura* and *D*. *barbatus*. The first of these is distributed on the mountains of Chiapas (Mexico) and from Guatemala to Costa Rica, whereas the latter two species are endemic to Mexico: *D*. *macroura* is distributed on the Trans-Mexican Volcanic Belt, the Sierra Madre del Sur and the Sierra Norte de Oaxaca, while *D*. *barbatus* is distributed on the Sierra Madre Oriental. Several authors have reported that these two species present overlapping ranges in the region of the Cofre de Perote and Pico de Orizaba volcanoes in Veracruz [1–4], (Fig 1) and possibly also in the Sierra Norte de Oaxaca (La Esperanza and Ixtlán, Oaxaca; [5]).

In addition to the sympatric area (overlapping distribution) between *D*. *macroura* and *D*. *barbatus*, they are sister species [6] and both are ecologically similar. For example, both species inhabit pine, pine-oak and cloud forest, although *D*. *macroura* has a higher preference for pine forests compared to *D*. *barbatus* that inhabits primarily cloud forests [1, 7, 8, 9]. Both species are terrestrial, gregarious, mate in early spring, and nest on the ground (laying four to six eggs), and both parents help care for the young and apparently remain paired throughout the breeding season [1]. The territorial calls of the two species are very strong and repetitive, but clearly differ in the number of notes [3, 10, 11]. Vocalizations are regularly emitted in the mornings or afternoons [8, 12]. However, both species are secretive and difficult to observe in the field, which has hampered their study and generated some uncertainty or lack of knowledge regarding their biology, taxonomy and behavioral traits [1, 5, 12, 13, 14]. This, coupled with a historical accumulation of occurrence records (many of which are based on questionable specimen labels, unsupported visual records, shallow bibliographical searches and non-systematic fieldwork), has had a major impact on the correct delimitation of their ranges [1, 2, 13, 14]. For example, according to the International Union for Conservation of Nature [15], *D*. *barbatus* is categorized as “Vulnerable”, while *D*. *macroura* is in the lower category of “Least Concern”, based on its apparently widespread distribution.

Distributional overlap between closely related bird species is often thought to be relatively rare [16, 17], perhaps because competitive interactions prevent the related species from expanding their distributions and overlapping in distribution [18, 19], resulting in allopatric (species formed in geographic isolation) or parapatric (those with adjacent but not overlapping distribution areas) speciation. Previous studies have analyzed the distribution of the genus *Dendrortyx* [9, 14]. For instance [9], evaluated the environmental and geographical distribution of *D*. *barbatus*, suggesting that the Santo Domingo River in northern Oaxaca represents the southern limit of its range, thus implying the absence of a sympatric area with *D*. *macroura*. On the other hand, [20] detected ambiguity in the information from the records of *D*. *macroura* in the central region of Veracruz (i.e. specimens collected–apparently–in Veracruz but with no collection date or collector name; and records in the literature), with no evidence such as specimens or photos in existence. However, there still remains a doubt about the potential area of sympatry between the two species and the reported overlap between their distributions therefore requires investigation.

The base of the Hutchinson’s niche definition is a *n*^-th^ dimensional space that represents the set of both environmental (e.g. temperature, humidity, altitude, etc.) and biotic (e.g. type of food, shelter sites for nesting, presence of other species, etc.) variables where a population can survive without immigration [21]. This is the basis of the different algorithms that generate Ecological Niche Models (ENM). Thus, the correlative combination of species records with environmental variables [22, 23] allows the identification of possible climatic areas where the distribution range of a species is present. When two species share similar ecological requirements in a given geographic area, they could theoretically compete for resources. Limitation of these resources could thus lead to exclusion of one of the two species, according to the principle of competitive exclusion [18, 19].

Many reference volumes suggest that *D*. *macroura* and *D*. *barbatus* share a narrow overlap in distribution [1–5]. To investigate the veracity of this suggestion, we visited museums to assess specimen localities and conducted fieldwork within the region of possible overlap. We also performed an ENM to determine the existence of appropriate climatic conditions for the potential presence of both species in this area, adding another source of information to dispel any remaining doubts about the existence of such area of sympatry. Considering the controversy regarding the overlapping area between these two species, in this study, we analyze new information compiled from this area and explore three hypotheses that could explain the long-standing beliefs about the distributional limits of these wood partridges: 1) an error in the single existing historical record; 2) a possible local extinction event or the presence of the species but at very low densities and 3) the past existence of interspecific competition that has since been resolved under the principle of competitive exclusion. Finally, we propose an alternative geographic boundary for the current distribution of these species.

## Materials and methods

We compiled historical and recent occurrence information pertaining to *D*. *macroura* and *D*. *barbatus* in Mexico based on: i) historical records reported in the literature, ii) queries of digital databases of available specimen and observational records, iii) examination of specimens deposited in ornithological collections in Mexico and the United States, and iv) fieldwork throughout the known range of both species:

### Historical records from literature

We conducted an exhaustive search of the historical records of *D*. *macroura* and *D*. *barbatus* in the scientific literature, from their original type descriptions up to the present (Table 1).

**Table 1:** Historical published references of the distribution area or occurrences records of *D*. *macroura* and *D*. *barbatus*. *Authors that suggest the presence of the *D*. *macroura* in Veracruz (on the volcanoes Cofre de Perote or Pico de Orizaba).

	Author	Publication date
	*D*. *macroura*	
1	Jardine and Selby [24]	1828
2	Jardine [25]	1834
3	Sumichrast [26]	1875
4	Sumichrast* [27]	1882
5	Nelson [28]	1897
6	Nelson [29]	1900
7	Ogilvie-Grant [30]	1897
8	Salvin and Godman* [31]	1897–1904
9	Beristain and Laurencio* [32]	1898
10	Hellmayr and Conover* [33]	1942
11	Friedmann [34]	1943
12	Ridgway and Friedman* [35]	1946
13	Leopold* [1]	1959
14	Warner [10]	1959
15	Phillips [36]	1966
16	Johnsgard* [37]	1972
17	Johnsgard* [2]	1973
18	Collar et al.,* [13]	1992
19	Watson [38]	2003
20	Morales-Mávil and Aguilar-Rodríguez* [39]	2000
21	Chávez-León and Velázquez [8]	2004
22	Chávez-León et al., [40]	2004
23	Peterson et al., [41]	2004
24	Montejo and McAndrews* [42]	2006
25	Chávez- León [43]	2010b
26	Gallardo and Aguilar-Rodríguez* [44]	2011
	*D*. *barbatus*	
27	Mota-Vargas and Rojas-Soto [14]	2012
28	Mota-Vargas et al., [9]	2013

### Digital databases

We consulted all available specimen and observational records of *D*. *macroura* archived at the Global Biodiversity Information Facility database GBIF [45], in eBird of the Cornell Lab of Ornithology [46] and in the Atlas of the Birds of Mexico [47]. All of these sources contain detailed geographic information.

### Ornithological collections

In order to obtain first-hand information on collection localities and to confirm the proper identification of specimens, we visited eight ornithological collections; three in Mexico and five in the USA: 1) Museo de Zoología “Alfonso L. Herrera”, Facultad de Ciencias (MZFC), UNAM, Mexico City. 2) Colección Nacional de Aves, Instituto de Biología, UNAM (CNA-IBUNAM), Mexico City. 3) Collection at the Universidad Michoacana de San Nicolás de Hidalgo (UMSNH), Morelia, Michoacán. 4) the Moore Laboratory of Zoology (MLZ), Los Angeles, CA. 5) Western Foundation of Vertebrate Zoology (WFVZ), Camarillo, CA. 6) Museum of Vertebrate Zoology (MVZ), University of California, Berkeley, CA. 7) American Museum of Natural History (AMNH), New York, NY; and 8) the National Museum of Natural History (NMNH), Washington, D.C.

### Fieldwork

**Fig 2:**
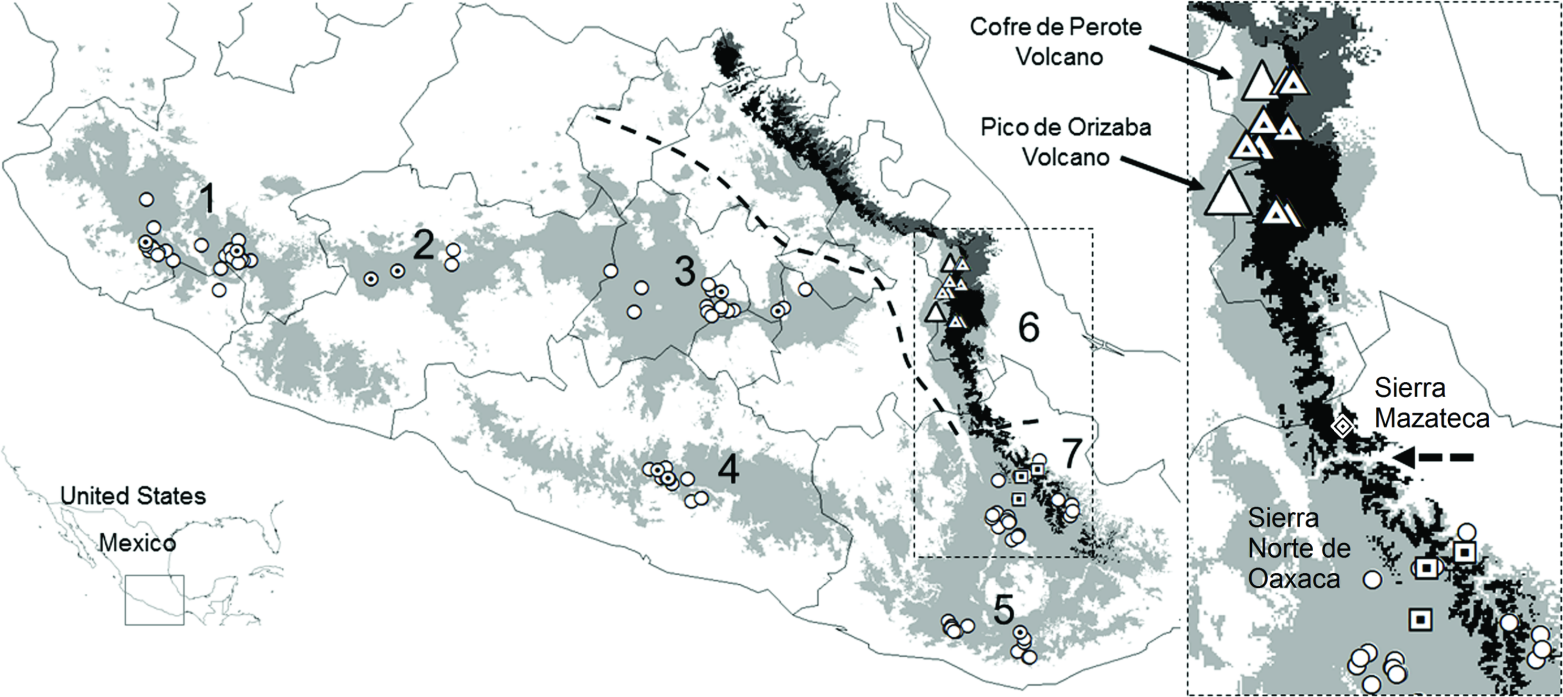
Predicted potential distribution of *D*. *macroura* (light gray), *D*. *barbatus* (dark gray), and overlapping area (black). The open circles represent historical records of *D*. *macroura*, the dotted circles represent the records where the presence of the species was currently confirmed in the field. The large white triangles represent the referred volcanoes in the text. The dotted triangles represent the records of *D*. *barbatus* with the absence of *D*. *macroura*; boxes represent field records of *D*. *macroura* with absence of *D*. *barbatus*. The diamond (in the Sierra Mazateca) represents the southernmost records of *D*. *barbatus* [9].

The distribution of *D*. *barbatus* was analyzed (including field work) in previous studies [9, 14]. In the case of *D*. *macroura*, we visited several localities in six states: 1) Jalisco (Parque Nacional Nevado de Colima and Estación Científica Las Joyas in the Reserva de la Biosfera Sierra de Manantlán), 2) Michoacán (Parque Nacional Barranca del Cupatitzio and Angahuan), 3) Estado de México (Parque Nacional Cumbres del Ajusco and Parque Nacional Izta-Popo Zoquiapan in the Trans-Mexican Volcanic Belt), 4) Guerrero (Omiltemi in the Sierra Madre del Sur), and 5) Oaxaca (Sierra de Miahuatlán). Furthermore, we focused our field surveys in two additional regions where both species were described as sympatric, with the aim of corroborating or rejecting their presence: 6) Veracruz (Cofre de Perote and Pico de Orizaba volcanoes) and 7) Oaxaca (three localities surveyed from La Esperanza to Santa María Jaltianguis in the Sierra Norte de Oaxaca; (Table 2, Fig 2). All localities visited were freely accessible to the public, so it was not necessary to request access permits.

**Table 2:** Regions and localities of fieldwork searching for *Dendrortyx* wood partridges in Mexico: longitude of transect (although the method was point playback, rather than transects) in km, sampling points (must indicate the total time surveyed in the points), number of presence records, m = D. macroura, b = D. barbatus, - = not sampled; date: month/year, and elevation (in masl). * Interviews. PNNC = Parque Nacional Nevado de Colima; RBSM = Estación Científica Las Joyas, Reserva de la Biosfera Sierra de Manantlán; PNBC = Parque Nacional Barranca del Cupatitzio; PNCA = Parque Nacional Cumbres del Ajusco; PN I-P = Parque Nacional Izta-Popo Zoquiapan.

Region	Locality	Km	Points	Records		Date	masl
				m	b		
1) Jalisco	PNNC	1.0	5	1	-	SEP/13	3370
	PNNC	1.0	5	3	-	SEP/13	3400
	PNNC	1.0	5	1	-	SEP/13	3400
	PNNC	1.8	9	8	-	SEP/13	3200
	RBSM	1.5	10	2	-	SEP/14	1995
	RBSM	0.5	2	2	-	SEP/14	1950
	RBSM	1.0	5	2	-	SEP/14	1943
	RBSM	1.5	6	2	-	SEP/14	1970
2) Michoacán	PNBC	1.5	10	11	-	SEP/13	2000
	PNBC	1.5	10	4	-	SEP/13	2050
	PNBC	1.5	10	6	-	AUG/14	1995
	PNBC	1.5	10	4	-	AUG/14	2000
	Angahuan	1.7	12	3	-	SEP/13	2600
3) Estado de México	PNCA	1.0	5	2	-	OCT/14	3230
	PN I-P	0.5	3	7	-	OCT/14	3150
4) Guerrero	Omiltemi	1.0	5	3	-	JAN/12	2300
	Omiltemi	1.5	6	4	-	JAN/12	2500
	Omiltemi	0.5	3	1	-	OCT/14	2250
	Omiltemi	1.0	5	1	-	OCT/14	2320
5) Oaxaca Sierra de Miahutlán, Oaxaca	San José del Pacífico	1.0	5	3	-	SEP/15	
	Sum	23.5	131	70			
Mean frequency of records:		2.98 records/km					
6) Oaxaca Sierra Norte de	Santa María Jaltianguis (5 km from Ixtlán) *	0.5	2	3	0	JAN/12	2560
	San Pedro Yolox	1.0	4	2	0	JAN/12	2460
	San Pedro Yolox	0.5	2	1	0	JAN/12	2180
	La Esperanza	1.0	4	1	0	JAN/12	1250
	La Esperanza	0.5	3	1	0	OCT/14	1770
Sum		3.5	15	8	0		
Mean frequency of records		2.28	records/km				
7) Veracruz	Cofre de Perote						
	Cosautlan*	0.5	2	0	1	MAY/13	1300
	Quimixtlan-Chilchotla	1.0	4	0	2	MAY/13	1970
	Francisco I. Madero	1.0	5	0	2	MAY/13	2455
	Ixhuacán*	1.0	4	0	2	MAY/13	2050
	El Zapotal	10.0	15	0	10	SEP/14	1800–2900
	Pico de Orizaba						
	Monte Blanco*	1.0	7	0	5	APR/13	1350
	Tetla-Xocotla*	2.0	7	0	6	APR/13	2050
Sum		16.5	44	0	28		
Mean frequency of records		1.69	records/km				

Field trips were carried out on January 2012, and April and September 2013, August-October 2014, and September 2015, corresponding to the months of greatest vocalization activity [8]. Within each region, we identified sites with well-preserved vegetation and surveyed them by walking or driving along transects from 500 m to 2 km. We sampled points spaced approximately 200 m apart along these transects. Surveys were conducted between 0630–1100 h, and from 1830–2000 h. While surveying these areas, we used playbacks because this technique has been widely demonstrated as effective, since these species readily respond to auditory stimuli [5, 8, 12]. We played the song of one species for 30 seconds, waited 30 seconds to listen for bird responses and played the song again. This was repeated three times in a row, for a total playback time of three minutes [48]. Spontaneous responses (i.e. without the auditory stimulus) while walking or driving between sampling points, were corroborated immediately using the playback method and the corresponding data recorded. Responses and no-responses of either species were recorded and the coordinates of each record were taken using a Garmin XL12 GPS. In the five Mexican states with historical records of *D*. *macroura*, we played the territorial song of a male [49]. In Veracruz (Cofre de Perote and Pico de Orizaba volcanoes) and the Sierra Norte de Oaxaca, where the two species have been considered to be sympatric (Fig 2), we played the territorial song of both *D*. *macroura* and *D*. *barbatus* (the latter from a tape recorded in 1996 by J. Eitniear in Coatepec, Veracruz). Each species is recognizable from their territorial songs, which are clearly different. We used a CD player and an amplifier to transmit the songs. At each sampling point, the amplifier was oriented in different directions in order to increase the chances of obtaining a response from individuals. Moreover, in those localities of reported sympatry, we also conducted eight unstructured interviews with groups of hunters and farmers (of 6–10 people each). We showed them pictures and played territorial songs of both *D*. *macroura* and *D*. *barbatus* in order to obtain information about their presence in the region (Table 2). The interviews were casual informal conversations; while we were working in the field and found a group of hunters or farmers, we approached them and talked as we walked together. The responses were recorded on field sheets.

### Ecological niche modeling

Finally, we analyzed the geographic and ecological patterns of these two species. We used the Genetic Algorithm for Rule-set Production (GARP) method for ENM, which is an artificial intelligence algorithm that works in an iterative way based on rules (atomic, logistic regression, and ranges). The GARP is a correlative model that uses occurrence records in combination with digital environmental layers [22]. For most species, there are insufficient occurrence records and they are frequently slanted by the accessibility of the sites [50]. As an alternative, ENM [51, 52], allows the generation of the species ecological niche based on a set of individual records of a species (with latitude–longitude data) that is related to the environmental variables in such localities [53, 54]. Thus, one can predict the geographic distribution of a species, even in areas lacking specimens, by projecting the niche into the geographical space [55]. To characterize the environmental niches, we used the occurrence records of *D*. *macroura* and *D*. *barbatus* (see below), 19 bioclimatic variables (Table 3) obtained from the WorldClim Project [56] and three topographic variables from the Hydro 1k project, United States Geological Survey, USGS, [57]; all layers had a spatial resolution of 0.0083° (~1 km^2^). These two sets of data were combined for model performance in an ecological or statistical space, where they interact to produce ecological distributions [23, 58], which were then projected onto geographic space [59, 60].

**Table 3:** Environmental variables used for ecological niche modeling (ENM).

1	BIO1 = Annual average temperature
2	BIO2 = Average daily range (mean monthly (max temp—min temp))
3	BIO3 = Isothermality (P2/P7) (* 100)
4	BIO4 = Temperature seasonality (standard deviation * 100)
5	BIO5 = Maximum temperature of warmest month
6	BIO6 = Minimum temperature of coldest month
7	BIO7 = Annual temperature range (P5-P6)
8	BIO8 = Average temperature of wettest quarter
9	BIO9 = Average temperature of driest quarter
10	BIO10 = Average temperature of warmest quarter
11	BIO11 = Average temperature of coldest quarter
12	BIO12 = Annual precipitation
13	Bio13 = Precipitation in wettest month
14	Bio14 = Precipitation in driest month
15	Bio15 = Seasonality of precipitation (coefficient of variation)
16	BIO16 = Precipitation in wettest quarter
17	BIO17 = Precipitation in driest quarter
18	BIO18 = Precipitation in warmest quarter
19	BIO19 = Precipitation in coldest quarter
20	CTI = Topographic index (a function of upstream contributing area and slope that reflects the tendency to pool water)
21	SLOPE
22	Elevation = Meters above sea level

We used representative records from digital databases (separated by more than 1 km) of the distribution range of the species in order to generate and evaluate these models. To run the models, we used 100% of the data points available (71 points for *D*. *macroura* and 78 for *D*. *barbatus*). For each instance, we ran 100 models with a convergence limit of 0.01 and a maximum of 1000 iterations [61]. The final maps were edited using the program ArcView 3.2 [62]. We evaluated model performance using the area under the curve (AUC) of the Receiver Operating Characteristic (ROC). We also used the partial-area ROC approach, following [63]. We implemented this analysis in stand-alone software [64]. We used 18 records of *D*. *macroura* and 20 of *D*. *barbatus* (independent records to those used to generate the model) to evaluate the predictions of the models. Finally, we measured the geographic overlap between both species using a Geographic Information System [62].

## Results

We reviewed 26 articles containing records of *D*. *macroura* from the scientific literature; in 12 of these, the presence of this wood partridge is reported in the highlands of Veracruz, a first potential area of sympatry (Table 1). On the other hand, Aguilar-Rodríguez [5] reports the presence of *D*. *barbatus* in Puerto Soledad, in the Sierra Mazateca, and suggests the presence of this species from La Esperanza to Ixtlán, in the Sierra Norte de Oaxaca, a second potential area of sympatry. From digital databases, we obtained a total of 718 different records of *D*. *macroura* (339 records from GBIF [45], 149 from eBird [46] and 230 from the Atlas of the Birds of Mexico [47]. All records correspond to the distribution range of *D*. *macroura* (Fig 2). However, from all of these sources of information, we only obtained a single sighting record from the area of overlap with *D*. *barbatus*. This record was from Monte Blanco (in the Metlac River Basin) on the eastern slope of the Pico de Orizaba volcano in Veracruz [46].

**Fig 3:**
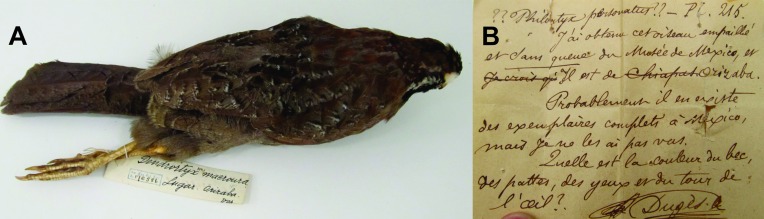
A) Specimen of *D*. *macroura* (No. Cat. AMNH 176586), with no collection date, nor collector identity, and a locality reference given as Orizaba, Ver. B) Letter signed by A. Dugès associated with the specimen of *D*. *macroura* (No. Cat. NMNH 124383), with no collection date or collector identity, and a locality reference given as Orizaba, Ver.

We examined a total of 148 specimens. Of these, 132 belonged to *D*. *macroura* and 16 to *D*. *barbatus*. Only two of the specimens of *D*. *macroura* were apparently collected in Orizaba, Veracruz; the first is deposited in the AMNH (No. Cat. AMNH 176586; Fig 3A) and the second in the NMNH (No. Cat. NMNH 124383), this latter specimen associated with a note (Fig 3B), which describes the manner in which it was obtained (see Discussion).

From our fieldwork, we confirmed the presence of *D*. *macroura* in five states of Mexico (Fig 2), where we obtained an average of 2.98 records/km surveyed. For the reported area of sympatry, we were able to corroborate the presence of only one of the two species: in the Sierra Norte de Oaxaca, we obtained an average of 2.28 records/km of survey for *D*. *macroura*; and in Veracruz (Cofre de Perote and Pico de Orizaba volcanoes), we obtained an average of 1.69 records/km of survey for *D*. *barbatus* (Table 2). However, we did not obtain responses for both species in any of the localities surveyed.

With respect to the interviews with people, none of the respondents (four groups of hunters and two groups of farmers) could confirm the presence of *D*. *macroura* in Veracruz (Cofre de Perote and Pico de Orizaba), nor did they identify the image or the territorial song of this species. However, when we showed them the picture and the territorial song of *D*. *barbatus*, at least one person from each of the interviewed groups identified the species with the common regional name of “chivizcoya”. The interviewees described characteristics of their vocal behavior and the red coloring of their legs and beak and many of them knew that they visit their bean and maize fields. Conversely, in the Sierra Norte de Oaxaca, two groups of hunters failed to identify *D*. *barbatus* but recognized the territorial song of *D*. *macroura* (Table 2).

**Fig 4:**
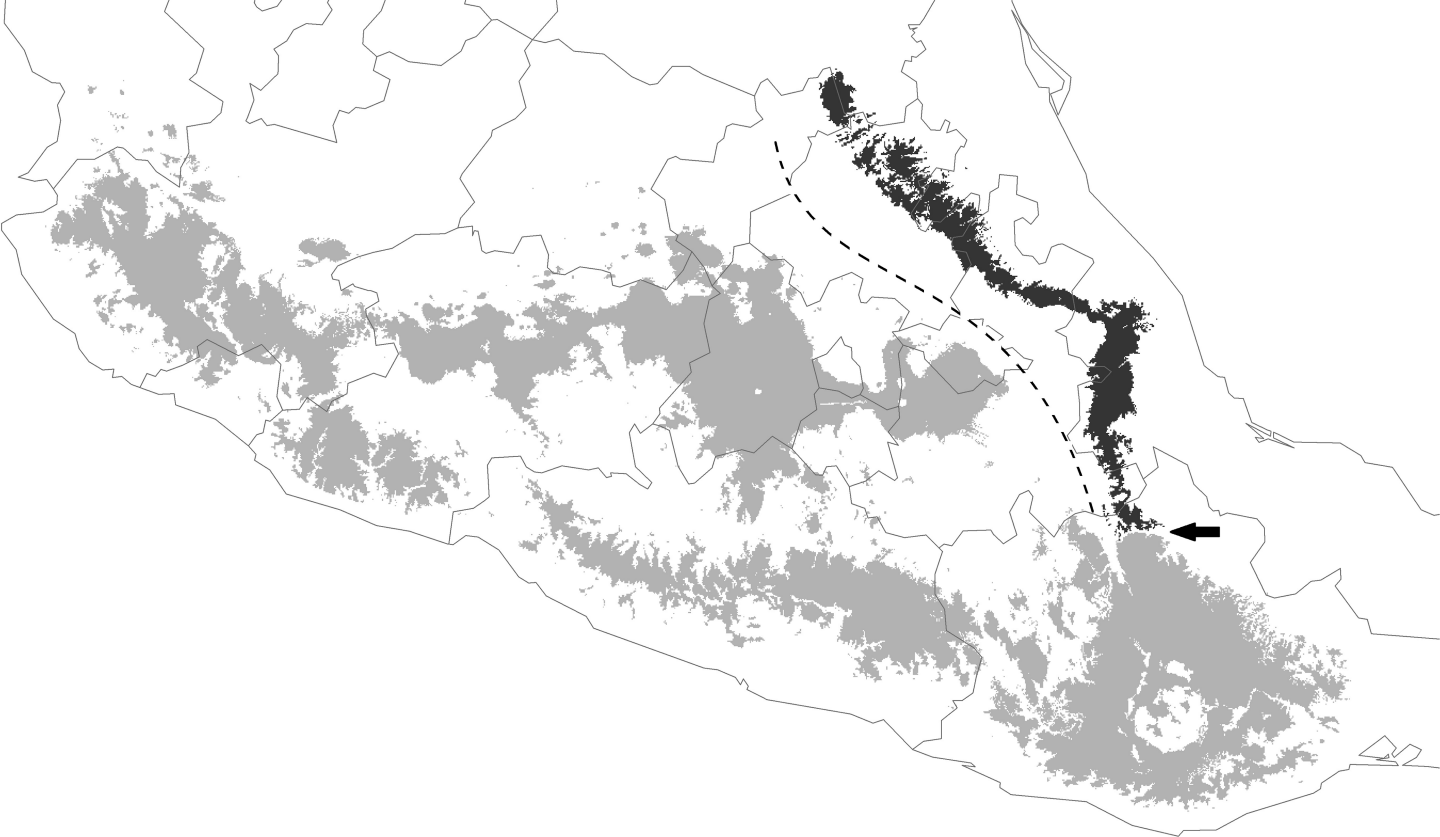
Proposed geographical distribution of *Dendrortyx macroura* (light gray) and *D*. *barbatus* (dark grey). The dotted line represents the proposed boundary between these two species. The arrow indicates the location of the Santo Domingo River that divides the Sierra Mazateca from the Sierra Norte de Oaxaca.

We generated an ENM for *D*. *barbatus* with an extension of 19,970 pixels and Partial ROC values significantly better than chance (ratio = 1.70, *P* = 0.044). For *D*. *macroura*, we obtained a model with 160,764 pixels and Partial ROC values significantly higher than chance (ratio = 1.69; *P* = 0.045). The area of geographical overlap between the two species was 9,918 pixels (1 pixel ~1 km^2^; Fig 2). Finally, we proposed the actual distribution and limit ranges for the species *D*. *macroura* and *D*. *barbatus* (Fig 4), according to our findings.

## Discussion

Our findings from the fieldwork do not support the records from the literature suggesting that *D*. *macroura* and *D*. *barbatus* are sympatric. The ENM suggests that both species may share a portion of their distributional range in Veracruz and the Sierra Norte de Oaxaca; however, specimen examination and analysis of historical records are both inconclusive in terms of supporting this putative sympatric area. What evidence has led many authors to report an area of sympatry? Below, we examine three alternative hypotheses that could explain the historically reported distribution pattern.

1) Long-tailed Wood-Partridge records on the Cofre de Perote and Pico de Orizaba volcanoes represent an historical mistake: *D*. *macroura* was described originally as *Ortyx macroura* by Jardine and Selby in 1828 [24]; they state about the holotype “The specimen we met with at the departure of Mr. Bullock’s Mexican curiosities…” without ever mentioning anything about the locality and collector’s name. Later, Jardine in 1834 [25], reported about this species: “We regret that nothing is known of its habit. It is a native of Mexico…” but provides no further information about its origin. Subsequently, Sumichrast in 1875 [26] reports the presence of *D*. *barbatus* in the Alpine region (1,500–3,500 masl) of Veracruz, but does not mention the presence of *D*. *macroura* in this area until 1882, when he reports the presence of both species in the Alpine region of Orizaba [27]. This was the first record of *D*. *macroura* in Veracruz; however, it is unclear what the basis of this record is, since the author did not collect any specimens in the region.

Different subspecies were described in the following years for *D*. *macroura*: *Dendrortyx oaxacae* (sensu [28]), *D*. *m*. *griseipectus*, *D*. *m*. *striatus* [28], *D*. *m*. *dilutus* [29], *D*. *m*. *diversus* [34] and *D*. *m*. *inesperatus* [36]. However, none of the distribution areas of the subspecies described corresponds to Veracruz State. Hellmayr and Conover [31] mentioned “*Dendrortyx macroura macroura* (Jardine and Selby, 1828) range Mountains about the Valley Mexico and highlands in the state of Vera Cruz, Mexico (sic)” and they refer to “material examined.—Mexico: Salazar, Sierra de la Cruz, alt. 10,000 feet, 2; Amecameca, 1; Vera Cruz, Mount Orizaba, 1.” However, the latter specimen does not appear in the database of the Field Museum of Natural History, Chicago [65]. Consequently, based on the above information, we must seriously question the presence of *D*. *macroura* in Veracruz.

Several other authors report the presence of *D*. *macroura* in Veracruz, but the information they use in support is based on the previous studies discussed above. For example, Beristain and Laurencio in 1898 [32] mentioned that the distribution area of *D*. *barbatus* and *D*. *macroura* as “Hab. Alpine region of Orizaba”. Salvin and Godman in 1897–1904 [31] refer to Sumichrast [27], while Ridgway and Friedman in 1946 [35] quote Beristain and Laurencio [32] and Salvin and Godman [31]. Leopold in 1959 [1] wrote about *D*. *macroura* and *D*. *barbatus*: “both species occur, for example, on Pico de Orizaba and Cofre de Perote.” However, Leopold did not list any collected specimens, visual, or vocalization records of *D*. *macroura* in Veracruz. Furthermore, based on his travel maps [1], we found no sampling points around Cofre de Perote nor Pico de Orizaba. Reports of the overlapping area by Leopold [1] are most likely based on the references available at that time. Despite the fact that the map locations of the fieldwork of A. S. Leopold in Mexico [1] showed the routes of travel, camps and collecting stations, in the central region of Veracruz (area of the volcanoes Cofre de Perote and Pico de Orizaba) Leopold had no collecting camps that could have confirmed the presence of *D*. *macroura* in the apparent area of sympatry.

Johnsgard [2] mentioned the distribution area of *D*. *macroura* as “…highlands of Mexico, from Michoacán and Veracruz, south to Oaxaca”, but this is also likely to be based on the information available at the time. Collar et al., [13] stated: “The species (*D*. *barbatus*) is thought to be sympatric with Long-tailed Wood-Partridge (*D*. *macroura*) in a few areas such as Pico de Orizaba and Cofre de Perote” and they refer to “Salvin and Godman 1897–1904, Leopold 1959, Johnsgard 1988.” In all of the previous publications, sympatry in the two species of *Dendrortyx* is reported second-hand and is not supported by any specimens.

Montejo Díaz [46] and Morales-Mávil and Aguilar-Rodríguez [39] posted observational records of *D*. *macroura* during short field visits to Pico de Orizaba (Monte Blanco) and Cofre de Perote, Veracruz, respectively. However, these two records are visual and this species is very difficult to observe, so this evidence cannot be regarded as definitive because there is no collected or photographic material to contrast. Moreover, in our fieldwork we did not obtain any records of *D*. *macroura* in the aforementioned localities, even using playback, which is a more trustworthy method. On the other hand, Aguilar-Rodríguez [5] suggests the possibility of *D*. *barbatus* presence in La Esperanza and Ixtlán, south of the Santo Domingo river in the Sierra Norte de Oaxaca; however, once again, we did not obtain records of *D*. *barbatus* in the region (Table 2), nor indications of the presence of this species in the area from other studies [9, 66].

We are left with the original specimen data as the only solid evidence of the reported range overlap. Neither of the two specimens of *D*. *macroura* apparently collected in Orizaba, Veracruz (AMNH Cat. No. 176586 and NMNH Cat. No. 124383; Fig 3) have a date or name of the collector. The second specimen even has a note in French that says “J’ai obtenu cet oiseau empaillé et dans queue du musée de Mexico, et il est de Orizaba. Probablement il en existe des exemplaires complets à Mexico, mais je ne les ai pas vas. Quelle est la couleur du bec, des pattes, des yeux et du tour de l’aile?” (in English: “I obtained this specimen from a museum shelf in Mexico, it is from Orizaba. There are probably more complete specimens in Mexico, but I have not seen them. What is the color of beak, legs, eyes, and wing?” signed by A. Dugès (Fig 3), a French-Mexican collector of plants, insects, and reptiles). To make it even more dubious, in the note of Dugès, the word Chiapas is crossed out before writing Orizaba. The *D*. *macroura* specimens collected in Veracruz would be the key evidence to support the reported presence of this bird in Veracruz; however, the information about these specimens is clearly very limited and certainly doubtful.

Despite the extensive sampling effort during our fieldwork, we did not find evidence of sympatry between these two species. Moreover, during interviews, we found that the species are locally conspicuous, but people interviewed in Veracruz failed to recognize either the image or the vocalization of *D*. *macroura*. Furthermore, personal communications from experienced ornithologists who work in the central region of Veracruz (e.g. Bernardino Villa, Fernando González, Leonel Herrera, Rafael Rodríguez, Robert Straub and Román Díaz), as well the literature [67] and Checklists of Veracruz [68], do not report the presence of *D*. *macroura* in Veracruz.

2) Are the reported literature records the result of a recent local extinction? or do both *Dendrortyx* wood partridges coexist at low densities? If *D*. *macroura* was present in the central part of the State of Veracruz, it could be locally extinct since specimen records of the species are very old, inconsistent and practically unsupported (e.g. [27]). Recent published records (e.g. [39]) were not from specimens, nor they were corroborated during our fieldwork, or in other recent studies in the state (e.g. [67]). Thus, the changes in land use and high rates of deforestation in Veracruz [69, 70] could have caused the extinction of *D*. *macroura* in Veracruz. It is known, however, that this species tolerates some habitat disturbance [8, 40]. On the other hand, both *D*. *macroura* and *D*. *barbatus* are considered “rare” species because of the difficulty of observing them directly in the field [1]. Our thorough searches using effective techniques to detect both species failed for only one of them. Our failure to detect *D*. *macroura* during our systematic surveys does not mean that it does not inhabit the region of central Veracruz; it may do so at very low densities. However, on the basis of current evidence, we believe that this last hypothesis is unlikely.

3) A historic coexistence of two species of *Dendrortyx* was resolved via competitive exclusion: the ENM generated from point records of *D*. *macroura* and *D*. *barbatus* suggest that there is an area of environmental and geographical overlap precisely in the center of Veracruz and the Sierra Norte region of Oaxaca (Fig 2). However, the models represent only a potential distribution based on climate predictors and do not explicitly consider biotic factors, accessibility or dispersal capacity; which act to determine the “true” area of distribution of a species [71, 72]. If at any historic time the two species were present in the same region, we could infer that competition would have occurred between these two closely-related species (which has been discussed extensively from the original competitive exclusion principle proposed by Gause [18] that would produce niche differentiation [21]). If, however, there is no such differentiation, or if it is precluded by the habitat, then one competing species will eliminate or exclude the other [73]). Complete competitors cannot coexist since ecological differentiation is the necessary condition for coexistence [19]. Complete ecological overlap is impossible and niches usually overlap only partially, with some resources being shared and others being used exclusively by each organism unit [74]. Thus, a possible explanation for the absence of one of the two species in “sympatric area” is the exclusion of one of the species from the conflict zone.

Three alternative explanations for this area of distribution overlap are likely; however, given the ambiguity of the information found in the literature, the lack of data from the two *D*. *macroura* specimens reportedly collected in Orizaba, Veracruz, and the lack of records found of either species during our fieldwork in the area of apparent sympatry, we can suggest that our crumble analyses do not support the existence of an area of sympatry between *D*. *macroura* and *D*. *barbatus* at all. We therefore consider that our data can instead be used to propose a revised distribution for both species that corrects what is most likely an error perpetuated over time by the repetition of dubious evidence.

Bindford [66] suggested “The canyon of the Rio Santo Domingo, with its tropical evergreen forests and arid tropical scrub, might separate populations of pine-oak birds in the Sierra de Juárez (Sierra Norte de Oaxaca) from those of the Sierra de Huautla (Sierra Mazateca sensu [75]), eastern Puebla, and west central Veracruz.” Based on this important statement, and the distribution limits of other species, for example amphibians such as *Pseudoeurycea bellii* [76], reptiles *Ophryacus smaragdinus* [77] and mammals *Peromyscus aztecus* [78], we propose to update and define the ranges for the species *D*. *macroura* and *D*. *barbatus* to the area illustrated in Fig 4. The closest area where the two species can occur (highlighted on the rectangular inset of Fig 2) considers the Santo Domingo River and the arid region of the western slope of the Sierra Madre Oriental, as the distribution limit between the two species, since it is at this point that the two distributions reach their northern and southern limits, respectively.
